# Light Trapping in Single Elliptical Silicon Nanowires

**DOI:** 10.3390/nano10112121

**Published:** 2020-10-25

**Authors:** Wenfu Liu, Yinling Wang, Xiaolei Guo, Jun Song, Xiao Wang, Yasha Yi

**Affiliations:** 1 School of Mechanical and Energy Engineering, Huanghuai University, Zhumadian, Henan 463000, China; wangyinling@huanghuai.edu.cn (Y.W.); guoxiaolei@huanghuai.edu.cn (X.G.); songjun@huanghuai.edu.cn (J.S.); 2 Integrated Nano Optoelectronics Laboratory, University of Michigan, Dearborn, MI 48128, USA; xwa@umich.edu; 3 Energy Institute, University of Michigan, Ann Arbor, MI 48109, USA

**Keywords:** silicon, single nanowires, elliptical cross-section, absorption, photocurrent

## Abstract

Light trapping in single nanowires (NWs) is of vital importance for photovoltaic applications. However, circular NWs (CNWs) can limit their light-trapping ability due to high geometrical symmetry. In this work, we present a detailed study of light trapping in single silicon NWs with an elliptical cross-section (ENWs). We demonstrate that the ENWs exhibit significantly enhanced light trapping compared with the CNWs, which can be ascribed to the symmetry-broken structure that can orthogonalize the direction of light illumination and the leaky mode resonances (LMRs). That is, the elliptical cross-section can simultaneously increase the light path length by increasing the vertical axis and reshape the LMR modes by decreasing the horizontal axis. We found that the light absorption can be engineered via tuning the horizontal and vertical axes, the photocurrent is significantly enhanced by 374.0% (150.3%, 74.1%) or 146.1% (61.0%, 35.3%) in comparison with that of the CNWs with the same diameter as the horizontal axis of 100 (200, 400) nm or the vertical axis of 1000 nm, respectively. This work advances our understanding of how to improve light trapping based on the symmetry breaking from the CNWs to ENWs and provides a rational way for designing high-efficiency single NW photovoltaic devices.

## 1. Introduction

Single nanowire (NW) solar cells have increasingly attracted attention in recent years due to the following reasons [1,2,3,4,5,6,7,8]. Firstly, single NW solar cells can serve as nanoscale power sources integrated seamlessly with nanoelectronics. Secondly, results from single NWs can subsequently provide feedback for the design of new functional NWs. Finally, single NWs can assemble to form NW arrays, and results from single NWs can help understand the self-assembled NW-based solar cells [7,8,9,10,11,12,13,14,15,16].

Light trapping in single NW solar cells is very important for ensuring both high absorption and little photoactive material, which can enhance the absorption by increasing the light path length within the cell while it is possible to use less material in the cell [17,18,19,20,21,22,23,24,25,26,27]. Therefore, light trapping is an effective way to improve light absorption and enhance the photoelectric conversion efficiency of single NW solar cell. It is well known that the strong interaction between the incident light and a single NW has been applied to dramatically increase light trapping due to the leaky mode resonances (LMRs) [28,29,30]. However, the overall light-trapping performance of a single NW is still far below expectations owing to the narrow resonant peaks.

Therefore, a great variety of strategies have been employed to improve light-trapping ability. It has been shown that the light-trapping performance could be readily engineered by controlling the size, geometry and orientation of the NWs [31,32,33,34,35,36,37]. Our previous studies [25,38,39] showed that the light-trapping performance could be further improved by introducing a non-absorbing dielectric shell (or graded dual shells) as the antireflection coating, which was experimentally and numerically demonstrated in the recent studies [40,41,42,43,44]. Recently, some new strategies have been implemented to improve the light-trapping capability of the NWs based on the symmetry breaking. For example, front (or rear)-opening crescent design [45,46], off-axial core-shell design [47,48], asymmetrical nanovoid design [33], partially capped design [49,50,51,52], nanocone design [53,54,55,56], inclined design [57,58,59] and disorder design [60,61,62,63,64]. Comparing with the circular NWs (CNWs), elliptical NWs (ENWs) provide more possibility to tune light trapping. It is worth noting that the elliptical nanostructures have been applied to improve light trapping in single ellipsoids [65,66], NW arrays [67], nanocone arrays [68] and nanohole arrays [61,69]. However, to the best of our knowledge, very few investigations based on the symmetry breaking from the CNW to ENW have been explored to improve light trapping in single NWs so far.

In this work, we carry out detailed investigations on the light-trapping effect of single ENWs. We demonstrate that the giant enhancement of the light absorption occurs when the ENWs replace the CNWs. The detailed analysis of the electric field, absorption mode profile and photogeneration rate shows that this enhancement is mainly attributed to the asymmetry breaking from the CNWs to ENWs. Specifically, the light path length can be increased by increasing the vertical axis and the LMR modes can be reshaped by decreasing the horizontal axis. Simulation results reveal that the photocurrent is significantly enhanced by 374.0% (150.3%, 74.1%) or 146.1% (61.0%, 35.3%) compared with that of the CNW with the same diameter as the horizontal axis of 100 (200, 400) nm or the vertical axis of 1000 nm, respectively.

## 2. Model and Methods

### 2.1. Model

**Figure 1:**
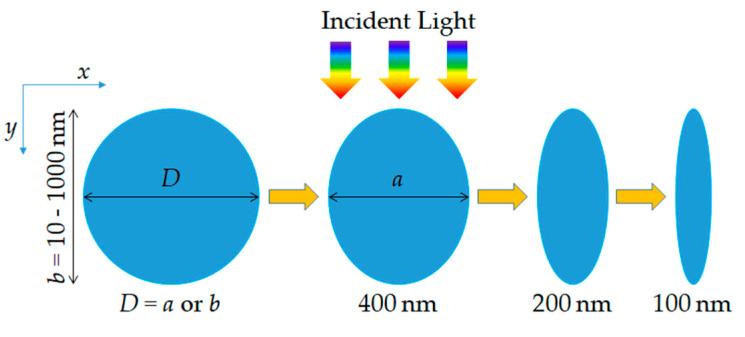
Schematics of the cross-section of a circle nanowire (CNW) and several elliptical nanowires (ENWs). The geometrical metrics used to describe the geometry of the ENWs, the horizontal (*x*) axis *a* and the vertical (*y*) axis *b*, are shown. Note here that three representative values of *a* are chosen to be 400, 200 and 100 nm, the range of *b* is from 10 to 1000 nm. The diameter *D* of the CNWs are chosen to be *a* or *b* for comparison, the material of the ENWs is set to be silicon as a representative semiconductor and the light illumination is perpendicular to the axis of the ENW from above.

Figure 1 schematically illustrates the cross-sectional views of a CNW and several ENWs. The horizontal (*x*) and vertical (*y*) axes of the ENWs are denoted by *a* and *b*. The horizontal (or vertical) axis is perpendicular (or parallel) to the light illumination direction, as shown using the colorful arrows in Figure 1. The values of *a* are chosen to be 400, 200 and 100 nm as the typical representative nanoscale size and the values of *b* are chosen to the range from 10 (oblate ENWs) to 1000 (prolate ENWs) nm. It should be noted that the CNWs are also shown for comparison, where the diameter *D* of the CNWs is chosen to be *b* to investigate the improved light trapping due to the reshaped LMRs by decreasing *a*, or *a* to investigate the improved light trapping owing to the increased light path length by increasing *b*. Silicon is chosen as a typical semiconductor material and its wavelength-dependent refractive index is adopted from the experimental data [70].

### 2.2. Methods

Numerical simulations are carried out by solving Maxwell’s equations based on the two-dimensional finite difference time domain (2D FDTD) method [71,72,73] by assuming that the length of the NWs is infinitely long, i.e., its length is far larger than the size of the cross-section, which can be referred to the work of Kim and co-workers for details [35,36,37,42]. In this simulation, the ENWs are illuminated perpendicularly by sunlight from the top, the wavelength range of the incident light is from 300 to 1100 nm with a step size of 5 nm considering solar radiation and the bandgap of silicon, the perfectly matched layers (PML) boundary conditions are applied to avoid any non-physical reflection with the boundaries, the total-field scattered-field (TFSF) method was adopted to ensure that a single NW interacts with an infinite plane wave. Also, the minimum cell size of the FDTD mesh is set to from 0.05 to 5 nm corresponding to *b* (5 → 1000 nm) to guarantee the accuracy of the simulation results.

#### 2.2.1. The Normalized Electric Field (E_r_)

The normalized electric field (*E_r_*) can be defined as [49]:

(1) $$E_{r}=E/E_{0},$$

where *E* is the electric field of the ENWs, which is obtained by FDTD numerical simulation, and *E*_0_ is the electric field of the solar incident light, respectively.

#### 2.2.2. The Absorption Mode Profile (P_abs_)

The wavelength-dependent absorption mode profile (*P*_abs_) calculated from the Poynting theorem can be expressed as [44,51,56,74]:

(2) $$P_{abs}=\frac{1}{2}\omega \varepsilon^{\prime \prime }{\left|E\right|}^{2},$$

where *ω* is the angular frequency of the incident light and ε” is the imaginary part of the permittivity of silicon, respectively.

#### 2.2.3. The Absorption Efficiency (Q_abs_)

To qualify the light-trapping performance of the ENWs, we define the absorption efficiency (*Q*_abs_) as [44,51,56,74]:

(3) $$Q_{\mathrm{abs}}=C_{\mathrm{abs}}/C_{\mathrm{geo}},$$

where *C*_geo_ is the projected area of the ENWs and *C*_abs_ is the absorption cross-section per unit length obtained by,

(4) $$C_{\mathrm{abs}}=\frac{\iint P_{abs}dxdy}{I_{0}}=k_{0}{\varepsilon^{\prime \prime }}_{r}\iint {\left|E_{r}\right|}^{2}dxdy,$$

where *k*_0_ is the wave vector in air,${\varepsilon^{\prime \prime }}_{r}$is the imaginary part of the relative permittivity of silicon, *x* and *y* are the coordinate axes shown in Figure 1, and *I*_0_ is the solar incident light intensity expressed as [44,51,56,74]:

(5) $$I_{0}=\frac{1}{2}c\varepsilon_{0}{\left|E_{0}\right|}^{2},$$

(6) $${\varepsilon^{\prime \prime }}_{r}=\varepsilon^{\prime \prime }/\varepsilon_{0},$$

where *c* is the speed of light and ε_0_ is the permittivity in air, respectively.

#### 2.2.4. The Photogeneration Rate (G)

The spatially dependent photogeneration rate (*G*) is readily calculated by [26,75]:

(7) $$G=\int_{300}^{1100}\frac{P_{abs}}{\hbar \omega }d\lambda =\int_{300}^{1100}\frac{\varepsilon^{\prime \prime }{\left|E\right|}^{2}}{2\hbar }d\lambda ,$$

where *ħ* is the reduced Planck’s constant and *λ* is the wavelength of the incident light. Note that when using Equation (7), each photon absorbed in ENW contributes to the photocurrent without considering recombination losses.

#### 2.2.5. The Ultimate Photocurrent (J_ph_)

The overall light-trapping performance is evaluated using the ultimate photocurrent (*J*_ph_) calculated by:

(8) $$J_{\mathrm{ph}}=\frac{q}{C_{geo}}\iint Gdxdy=q\int_{300}^{1100}\Gamma (\lambda )Q_{abs}(\lambda )d\lambda ,$$

where *q* is the elementary charge and Γ is the AM1.5G standard solar photon flux density spectrum. It should be noted here that 100% collection efficiency is assumed, which has been widely employed to evaluate the ultimate photocurrent [29,75].

#### 2.2.6. The Photocurrent Enhancement Factor (PEF)

The photocurrent enhancement is evaluated by employing the photocurrent enhancement factor (PEF) using the relation:

(9) $$\mathrm{PEF}=\left(J_{\mathrm{ph},\mathrm{ENWs}}-J_{\mathrm{ph},\mathrm{CNWs}}\right)/J_{\mathrm{ph},\mathrm{CNWs}},$$

where *J*_ph,ENWs_ and *J*_ph,CNWs_ are the photocurrent density for the ENWs and CNWs, respectively.

## 3. Results and Discussion

### 3.1. Light-Trapping Mechanism in Single Elliptical Nanowire (ENW)

To understand the light-trapping mechanism responsible for the improved absorption of the ENW, we investigate the absorption efficiency (*Q*_abs_), ultimate photocurrent (*J*_ph_), normalized electric field (*E_r_*), absorption mode profile (*P*_abs_) and photogeneration rate (*G*), respectively. Note here that *a* = 200, *b* = 500 nm and *D* = *a* = 200 or *D = b* = 500 nm denotes the cases of the CNW with the same *a* or *b* for comparison, respectively.

#### 3.1.1. The Absorption Efficiency (Q_abs_)

**Figure 2:**
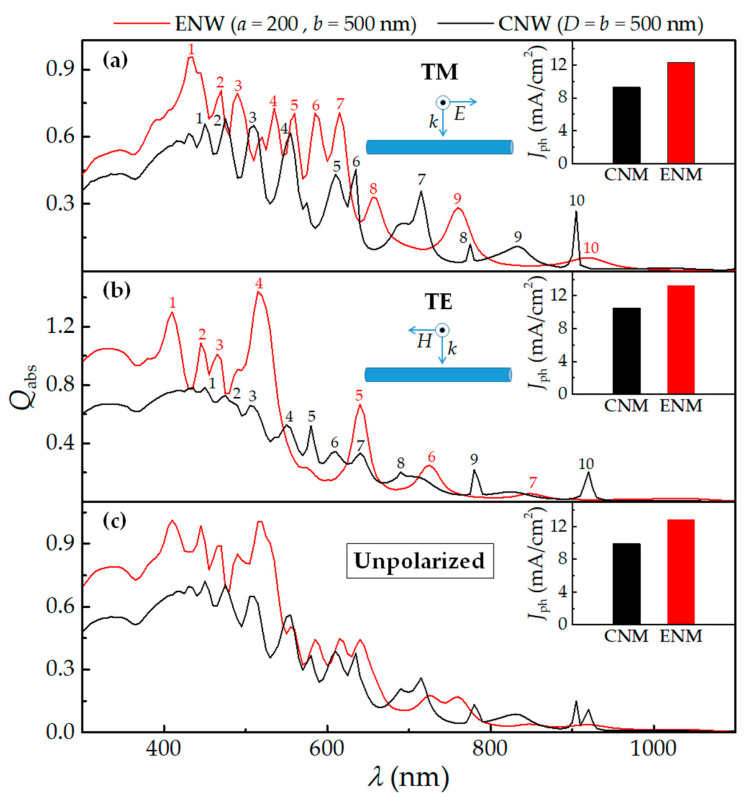
Absorption efficiency (*Q*_abs_) of the CNW and ENW as a function of wavelength (*λ*) for (**a**) transverse-magnetic (TM), (**b**) transverse-electric (TE) and (**c**) unpolarized light illumination, respectively. The insets at the top center illustrate the illumination geometries for (**a**) TM (electric field parallel to the axis of the ENW) and (**b**) TE (electric field perpendicular to the axis of the ENW) light illumination, respectively. Also, the insets at the top right corner show the ultimate photocurrent (*J*_ph_) of the CNW and ENW for (**a**) TM, (**b**) TE and (**c**) unpolarized light, respectively. Note that *a* = 200 and *D* = *b* = 500 nm.

To quantitatively characterize the light-trapping performance of the ENW in comparison with the CNW, we first examine the absorption spectra obtained by Equation (3). In Figure 2, we show *λ-*dependent *Q*_abs_ spectra of the ENW with *a* = 200 and *b* = 500 nm and the CNW with *D* = *b* = 500 nm under normally-incident transverse-magnetic (TM), transverse-electric (TE) and unpolarized light, respectively. It is important to emphasize that the unpolarized illumination, like sunlight, is calculated by averaging TM and TE light illumination. TM and TE lights are shown in the inset in the top center of Figure 2a and Figure 2b, respectively. It is clear that *Q*_abs_ of the ENW is much bigger than that of the CNW in the short-wavelength range of *λ* < *λ*_cTM_ ~ 675, *λ* < *λ*_cTE_ ~ 545 or *λ* < *λ*_c_ ~ 670 nm for TM, TE or unpolarized light (except for several narrow peaks, for example, *λ* = 555 nm for unpolarized light), which can result in a significant photocurrent enhancement since the *Q*_abs_ spectra of the CNW well matches the solar spectrum according to Equation (8). In comparison, the light absorption of the ENW seems to be comparable in the long-wavelength range of *λ* > *λ*_cTM_, *λ* > *λ*_cTE_ or *λ* > *λ*_c_, which can lead to a little contribution to the photocurrent enhancement. Note that *λ*_cTM_, *λ*_cTE_ and *λ*_c_ are the characteristic wavelengths, below which the light absorption is always enhanced and can be readily determined for a fixed *a*, *b* and *D*.

Moreover, the *Q*_abs_ spectra for TM light present some characteristics different from that for TE light. Specifically, compared to the *Q*_abs_ spectra of the CNW with *D* = *b* = 500 nm, the number of resonant peaks is increased (5 → 7) in the wavelength range of *λ* < *λ*_cTM_ due to the blue-shift of peaks and the light absorption is slightly improved for TM light, for example, 0.62 → 0.73 for the fourth peak, however, the number of resonant peaks is decreased (10 → 7) in the whole wavelength range and the light absorption is dramatically enhanced in the wavelength range of *λ* < *λ*_cTE_ at the same time, for instance, 0.53 → 1.44 for the fourth peak. Note here that some *Q*_abs_ values for TE light exceed unity, which is attributed to the fact that the absorption cross-section is greater than the physical cross-section. All in all, the absorption results indicate the great potential of the single ENW in improving light trapping due to the symmetry-broken structure from the CNW to ENW.

#### 3.1.2. The Ultimate Photocurrent (J_ph_)

To evaluate the light-trapping performance of the ENW for photovoltaic applications, we then calculated the ultimate photocurrent (*J*_ph_) according to Equation (8). For a direct comparison, in the insets of the upper right corner of Figure 1, we show *J*_ph_ of the ENW and CNW corresponding to *Q*_abs_ for TM, TE and unpolarized light illumination, respectively. It is observed that *J*_ph_ of the ENW is much bigger than that of the CNW with *D* = *b* = 500 nm. *J*_ph_ for TM, TE and unpolarized light illumination reaches 12.32, 13.24 and 12.78 mA/cm^2^, which is 32.5%, 26.6% and 29.4% higher than that of the CNW (9.30, 10.46 and 9.88 mA/cm^2^), respectively. It is worth noting that the photocurrent enhancement is mainly ascribed to the reshaped LMRs caused by decreasing *a* (that is *D* → *a*, here 500 → 200 nm) compared to the CNW with *D* = *b* = 500 nm, as discussed later. Moreover, *J*_ph_ can be significantly enhanced due to the increased light path length by increasing *b* (*D* → *b*, here 200 → 500 nm) compared with the CNW with *D* = *a* = 200 nm. *J*_ph_ is 50.2%, 82.9% and 65.5% higher than that of the CNW (8.20, 7.24 and 7.72 mA/cm^2^) [39] for TM, TE and unpolarized light illumination, respectively. The photocurrent results further indicate the huge potential of light trapping in single ENW for photovoltaic applications.

#### 3.1.3. The Normalized Electric Field (E_r_)

**Figure 3:**
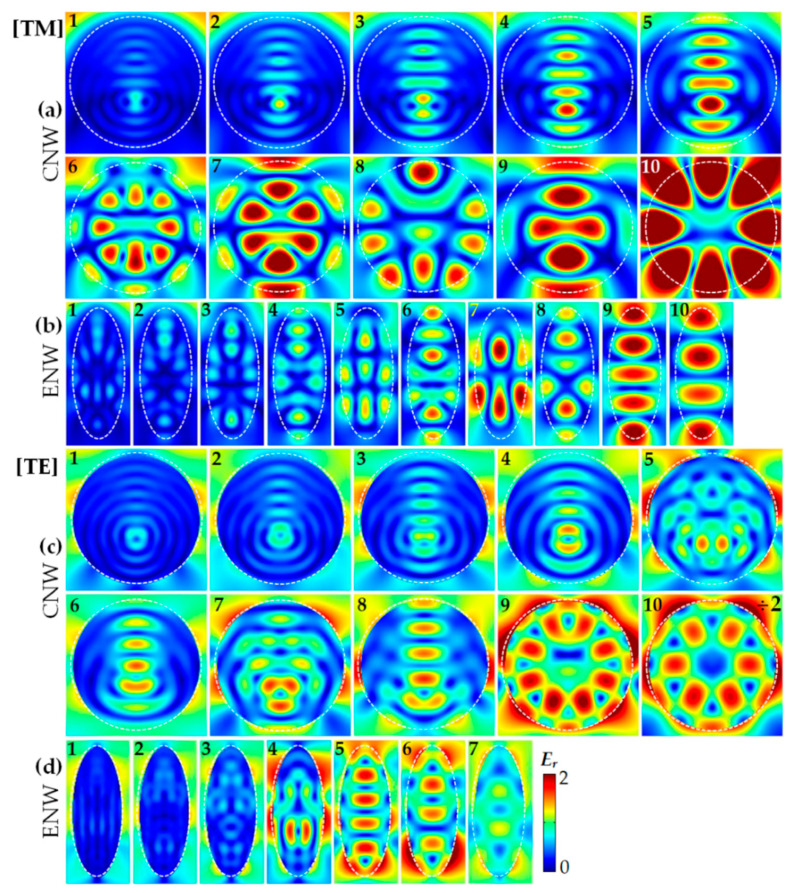
The representative normalized electric field (*E_r_*) profiles of the CNW and ENW at the absorption peaks indicated by Arabic numerals in Figure 2a,b: (**a**,**b**) for TM and (**c**,**d**) for TE light illumination (with an identical color scale); (**a**,**c**) for the CNW with *D* = *b* = 500 nm and (**b**,**d**) for the ENW with *a* = 200 and *b* = 500 nm, respectively. Note that the *E_r_* intensity in **c**(10) is properly shrunk for better illustration.

To understand the mechanism of light trapping in single ENW, we first examine the normalized electric field (*E_r_*) calculated by Equation (1). In Figure 3, we present the *E_r_* profiles of the CNW (*D* = *b* = 500 nm) and the ENW (*a* = 200 and *b* = 500 nm) corresponding to the positions denoted by Arabic numerals in Figure 2a,b under TM and TE light illumination, respectively. Note that Figure 3a,c show the *E_r_* profiles of the CNW for TM (*λ* = 450, 475, 510, 550, 610, 635, 715, 775, 835 and 905 nm) and TE (*λ* = 450, 475, 505, 550, 580, 610, 640, 690, 780 and 920 nm) light illumination, while Figure 3b,d show those of the ENW for TM (*λ* = 435, 470, 490, 535, 560, 585, 615, 655, 760 and 920 nm) and TE (*λ* = 410, 440, 465, 515, 640, 725 and 850 nm) light illumination, respectively.

It is observed that there are common characteristics of the *E_r_* profiles between the ENW and CNW. Firstly, the enhanced light trapping of the ENW is attributed to the excitation of the LMRs, likewise in CNW [28,29], which can confine light by multiple total internal reflections at the ENW/air interface when the wavelength of the incident light matches one of the LMRs supported by the ENW. The LMRs can be termed as TM*_ml_* or TE*_ml_*, where *m* and *l* describe the azimuthal mode number and the radial order of the resonances, respectively. For example, the *E_r_* profiles of the ENW in Figure 3b(10) and Figure 3d(7) show more characteristics of the TM_12_ and TE_11_ modes of the CNW, respectively. Secondly, both NW configurations show much higher *E_r_* intensities in the long- than in the short-wavelength range. Specifically, the *E_r_* intensities for both NW configurations are much higher in Figure 3a,b(5–10) than Figure 3a,b(1–4) for TM light, while the *E_r_* intensities of the CNW in Figure 3c(5–10) and the ENW in Figure 3d(4–7) are much bigger than those of the CNW in Figure 3c(1-4) and the ENW in Figure 3d(1–3) for TE light, respectively, which is attributed to the fact that${\varepsilon^{\prime \prime }}_{r}$is much smaller in the long- than short-wavelength range, resulting in a stronger resonance.

More importantly, there are many distinct characteristics of the *E_r_* profiles for both NW configures due to the symmetry breaking from the CNW to ENW. Firstly, the *E_r_* profiles of the CNW exhibit a large number of exact (for example, TM_51_ in Figure 3a(8) and TE_13_ in Figure 3c(9)) and approximate degeneracies (for example, TM_51_ in Figure 3a(10) and TE_13_ in Figure 3c(10)). In contrast, the *E_r_* profiles of the ENW exhibit different numbers, positions and modes of the resonant peaks between TM and TE light, indicating the higher tunability of light trapping in ENW than CNW. Secondly, the *E_r_* intensities at some resonant peaks are much larger only near the vertical axis of the CNW (for example, Figure 3a(1-5) for TM light and Figure 3c(1–4,6,8) for TE light), while those at all resonant peaks are much bigger inside the whole ENW owing to the excitation of more complex LMRs in the short-wavelength range (for example, Figure 3b(1–4) for TM light and Figure 3d(1-3) for TE light), indicating the more vital interaction of incident light with the ENW, leading to a more significant light trapping comparison with the CNW.

#### 3.1.4. The Absorption Mode Profile (P_abs_)

**Figure 4:**
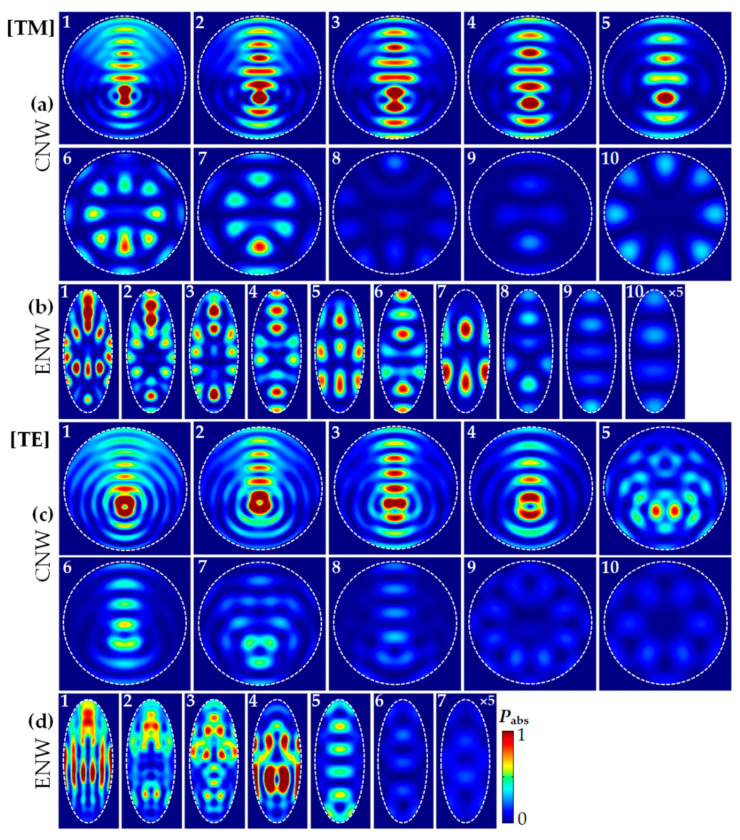
The representative normalized absorption mode profiles (*P*_abs_) for the CNW and ENW at the absorption peaks indicated by Arabic numerals in Figure 2a,b and Figure 3: (**a**,**b**) for TM and (**c**,**d**) for TE light illumination (with an identical color scale); (**a**,**c**) for the CNW with *D* = *b* = 500 nm and (**b**,**d**) for the ENW with *a* = 200 and *b* = 500 nm, respectively. Note that *P*_abs_ in **b**(10) and **d**(7) are properly magnified for better illustration.

To further understand the physics behind light trapping in single ENW, we then examine the absorption mode profile (*P*_abs_) calculated by Equation (2). In Figure 4, we present the normalized *P*_abs_ of the CNW (*D* = *b* = 500 nm) and the ENW (*a* = 200 and *b* = 500 nm) corresponding to the same positions denoted by Arabic numerals in Figure 2 and Figure 3 under TM and TE light illumination, respectively. Note that Figure 4a,c show the normalized *P*_abs_ of the CNW for TM and TE light, while Figure 4b,d show those of the ENW, respectively. It is observed that both NW configurations exhibit much higher absorption in the short- and medium- than long-wavelength range. Specifically, the light absorption for both NW configurations is much higher in Figure 4a,b(1–7) than Figure 4a,b(8-10) for TM light, while the light absorption of the CNW in Figure 4c(1–7) and the ENW in Figure 4d(1-5) is much bigger than that of the CNW in Figure 4c(8–10) and the ENW in Figure 4d(6–7) for TE light, respectively, which is attributed to the fact that${\varepsilon^{\prime \prime }}_{r}$is much greater in the short- and medium- than long-wavelength range, leading to a stronger absorption in the short- and medium wavelength range.

It is worth noting that the match between${\varepsilon^{\prime \prime }}_{r}$and *E_r_* becomes another essential factor in evaluating the absorption in the specific wavelength according to Equation (2). For instance, although the *E_r_* intensities of the ENW at λ = λ_10_ = 920 nm for TM light is much larger, the corresponding${\varepsilon^{\prime \prime }}_{r}$is the smallest, which still leads to a lower absorption, while although those of the ENW at λ = λ_1_ = 410 nm for TM light is the smallest, the corresponding${\varepsilon^{\prime \prime }}_{r}$is much larger, which results in a more significant absorption. It is readily observed that the spatially localized absorption sites of the ENW in both ends of the horizontal axis are considerably increased compared to the CNW, and more absorption sites appear and fill in the whole ENW. Such enhanced strong light–matter interaction results in giant light trapping, as shown in Figure 2.

#### 3.1.5. The Photogeneration Rate (G)

**Figure 5:**
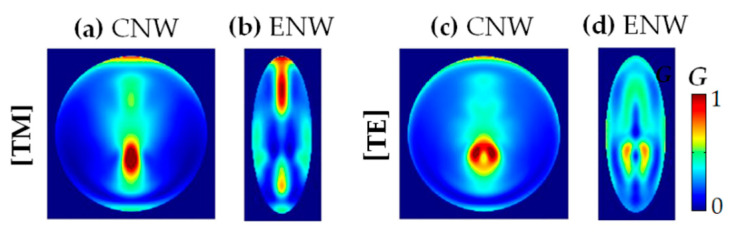
The normalized photogeneration rate (*G*) profiles for the CNW and ENW: (**a**,**b**) for TM and (**c**,**d**) for TE light illumination (with an identical color scale); (**a**,**c**) for the CNW with *D* = *b* = 500 nm and (**b**,**d**) for the ENW with *a* = 200 and *b* = 500 nm, respectively.

To further confirm the physical mechanism discussed above, we show the photogeneration rate (*G*) calculated by Equation (6). In Figure 5, we present the normalized *G* profiles of the CNW (*D* = *b* = 500 nm) and the ENW (*a* = 200 and *b* = 500 nm) for TM and TE light illumination. Note that Figure 5a,c show the normalized *G* profiles of the CNW for TM and TE light, while Figure 5b,d show those of the ENW, respectively. It is observed in Figure 5a,b that the absorption of the ENW is much stronger at both ends of the horizontal axis than that of the CNW for TM light. At the same time, it is observed in Figure 4c,d that the absorption of the ENW fills in the whole NW for TE light, consistent with the results discussed above. These results further demonstrate that this enhancement arises mainly from the excitation of more LMR modes caused by decreasing *a* compared to the CNW with *D* = *b*. In other words, the ENW can better interact with the incident light compared to the CNW, leading to more resonant absorption sites appearing and filling in the whole ENW.

### 3.2. The Light-Trapping Performance of Single ENWs

To verify that the improved light trapping is not just specific for the dimension discussed above, we calculate *Q*_abs_ and *J*_ph_ of the ENWs with *b* = 10 → 1000 nm and *a* = 400, 200 and 100 nm, and *Q*_abs_ and *J*_ph_ of the CNWs with *D* = *b* are investigated for comparison.

#### 3.2.1. The Absorption Efficiency of Single ENWs

**Figure 6:**
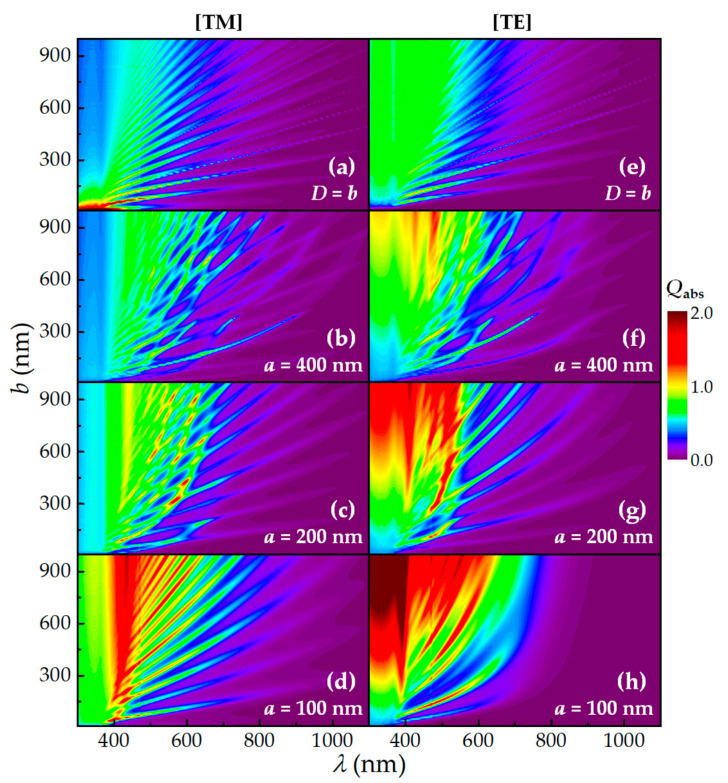
*Q*_abs_ versus *b* and λ for the CNWs and ENWs: (**a**–**d**) for TM and (**e**–**h**) for TE light illumination (with an identical color scale); (**a**,**e**) for the CNW with *D* = *b*; (**b**,**f**,**c**,**g**,**d**,**h**) for the ENWs with *a* = 400, 200 and 100 nm, respectively.

To evaluate the light-trapping performance of the ENWs for photovoltaic applications, we first investigate the effect of its geometrical parameters on the absorption efficiency calculated using Equation (3). In Figure 6, we show 2D contour maps of λ-dependent *Q*_abs_ as a function of *b* (10 → 1000 nm) for the ENWs with *a* = 400, 200 and 100 nm for TM and TE light illumination, respectively. Note that the *Q*_abs_ spectra of the CNWs with *D* = *b* is also shown for comparison. It is observed that the *Q*_abs_ spectra of the ENWs show the extensive wavelength tenability, and the light absorption of the NEWs with *b* > *a* is dramatically enhanced compared to that of the CNWs for both polarized lights.

On the one hand, the *Q*_abs_ spectra of the ENWs exhibit some characteristics similar to the CNWs. Firstly, the number of resonant peaks increases. For example, Figure 6d shows five and 11 peaks for the ENWs with *b* = 300 and 900 nm, respectively. Secondly, the peaks tend to red-shift with increasing *b*. For instance, in Figure 6d, the peak at near λ = 420 nm for the ENW with *b* = 300 nm can shift substantially to about λ = 800 nm for the ENW with *b* = 1000 nm. Finally, the resonant peaks in different ENWs may originate from entirely different resonances. For example, Figure 6d shows the peaks near λ = 600 nm for the ENWs with *b* = 300 and 600 nm.

On the other hand, the *Q*_abs_ spectra of the ENWs also show some characteristics different from the CNWs. Firstly, the light absorption of the CNWs is gradually weakened due to the weak LMRs with increasing *D*. In contrast, the light absorption of the ENWs is significantly enhanced with increasing *b* for various *a* for both polarized lights, which can be attributed to the reshaped LMRs due to the small size of the horizontal axis. Secondly, the *Q*_abs_ spectra of the ENWs with large *a* = 400 and 200 nm show some enhanced absorption sites owing to the superposition of resonant peaks, which may result from different LMR modes caused by the size difference between the horizontal and vertical axes. These enhanced absorption sites are greatly strengthened and tend to blue-shift with decreasing *a* = 400 → 200 nm, and then the resonant peaks for TM light exhibit similar peaks with the CNWs, while those for TE light present some new peaks utterly different from the CNWs with further deceasing *a* = 200 → 100 nm. For example, the number of resonant peaks decreases, and the peaks tend to blue-shift, but the light absorption is dramatically enhanced. Thirdly, with decreasing *a* = 400 → 100 nm, the light absorption is significantly enhanced in the short-wavelength range of λ < ~420 nm. Finally, The light absorption for TE light is much higher than that for TM light, resulting in a bigger photocurrent for TE light.

It is worth noting here that the light absorption near λ = 350 nm of the CNWs with small *D* (< 100 nm) is higher than that of the ENWs for TM light due to the excitation of the strongest TM_01_ and second strongest TM_11_/TE_01_ modes [29,38]; however, such small NWs are not practical for photovoltaic applications. In a word, these *Q*_abs_ spectra indicate that the light absorption of the ENWs with *b* > *a* can be significantly enhanced compared to that of the CNWs.

#### 3.2.2. The ultimate photocurrent of Single ENWs

**Figure 7:**
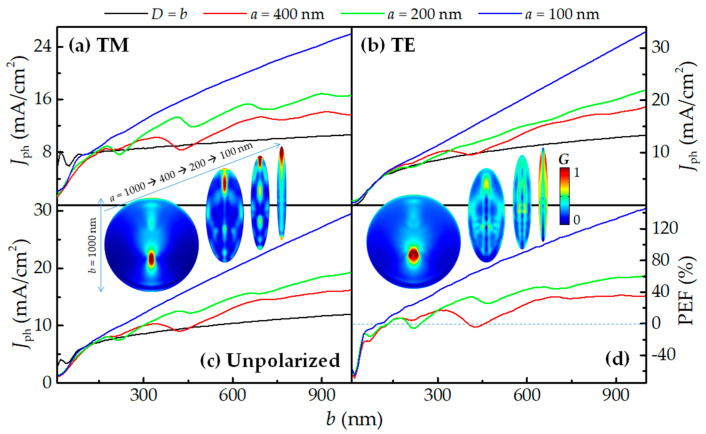
*J*_ph_ versus *b* of the CNWs with *D* = *b* and the ENWs with *a* = 400, 200 and 100 nm for (**a**) TM, (**b**) TE and (**c**) unpolarized light illumination, respectively. (**d**) The photocurrent enhancement factor (PEF) versus *b* of the ENWs compared to the CNWs. The insets illustrate the normalized *G* profiles of the CNWs with *D* = *b* = 1000 nm and the ENWs with *b* = 1000 nm and *a* = 400, 200 and 100 nm for TM and TE light illumination (with an identical color scale), respectively.

To evaluate the light-trapping performance of the ENWs for photovoltaic applications, we now investigate the influence of geometrical parameters on the ultimate photocurrent (*J*_ph_) calculated using Equation (8). In Figure 7a–c, we show *J*_ph_ as a function of *b* (10 → 1000 nm) of the ENWs with *a* = 400, 200 and 100 nm and the CNWs with *D* = *b* for TM, TE and unpolarized light illumination, respectively. It is shown that for *b* < *a*, with increasing *b*, *J*_ph_ of the ENWs with small *b* (<100 nm) is much smaller than that of the CNWs due to the excitation of the strongest TM_01_ and second strongest TM_11_/TE_01_ modes of the CNWs [29,38] and then can be slightly enhanced when *b* → *a* due to the superposition of resonant peaks, this enhancement is more evident for large than small *a*. More importantly, for *b* > *a* (except for *a* → *b*), with increasing *b*, *J*_ph_ of the ENWs is dramatically enhanced compared to the CNWs. Specifically, *J*_ph_ periodically increases for large *a* = 400 → 200 nm and linearly increases for small *a* = 100 nm.

In the insets of Figure 7, we show the normalized *G* profiles of a CNW and three ENWs for TM and TE light illumination, respectively. Note here that *D* = *b* = 1000 nm, and *a* = 400, 200 and 100 nm, respectively. As presented above, the absorption enhancement of the ENWs only occurs near the vertical axis, leading to the small photocurrent enhancement with increasing *b*. However, with the symmetry breaking from CNWs to ENWs (*a* = 1000 → 400 → 200 → 100 nm), the LMR modes are reshaped due to the size decrease of the horizontal axis, and the absorption enhancement sites better fill in the whole ENWs for both TM and TE lights, especially TE light.

Finally, we show in Figure 5c the photocurrent enhancement factors (PEFs) defined by Equation (9). It is readily observed that *J*_ph_ of the ENWs with *b* > *a* (except for *a* → *b*) is much larger than the CNWs. In particular, *J*_ph_ reaches 29.53, 19.32 and 16.23 mA/cm^2^ for *a* = 100, 200 and 400 nm, which is 146.1%, 61.0% and 35.3% much larger than that of the CNW with *D* = *b* = 1800 nm (12.00 mA/cm^2^) due to the reshaped LMRs caused by the small size of the horizontal axis and 374.0%, 150.3% and 74.1% much larger than that of the CNW with *D* = *a* = 100 (6.23 mA/cm^2^), 200 (7.72 mA/cm^2^) and 400 (9.32 mA/cm^2^) due to the increased light path length by the vertical axis, respectively.

## 4. Conclusions

In summary, we demonstrated the enhanced light trapping from the CNW to ENW. The influences of the geometrical parameters of the ENW on the light-trapping performance were numerically investigated. It was found that the elliptical cross-section can lead to significantly improved light trapping. The examination of the spatial profiles of the electric field, absorption mode and photogeneration rate revealed that the enhancement effect resulted from the symmetry-broken structure, which can simultaneously realize the increase of the light path length by the vertical axis and the reshaped LMRs by the horizontal axis. The simulation results showed that the photocurrent was significantly enhanced by 374.0% (150.3%, 74.1%) or 146.1% (61.0%, 35.3%) in comparison with that of the CNW with the same diameter as the horizontal axis of 100 (200, 400) nm or the vertical axis of 1000 nm, respectively. Therefore, such an elliptical nanowire can be applied to various semiconductors to improve light trapping and provides a promising approach for the future development of high-efficiency single NW solar cells.
